# MCPIP1 Controls Hybrid EMT and Tumor Stemness via the IL6/JAK2/STAT3 Axis in Pancreatic Cancer

**DOI:** 10.1002/cam4.71179

**Published:** 2025-08-28

**Authors:** Xihui Ding, Yingying Zheng, Min Liu, Fu Lai, Shiqi Liu, Qiuping Chen, Zihao Zhu, Huanzhong Liu, Xiaohui Li, Jinyong Xu, Rui Wang, Zhenhua Ren

**Affiliations:** ^1^ Department of Anatomy Anhui Medical University Hefei Anhui China; ^2^ Second Clinical Medical College Anhui Medical University Hefei Anhui China; ^3^ Department of Psychiatry Chaohu Hospital of Anhui Medical University Hefei Anhui China; ^4^ Anhui Provincial Key Laboratory for Brain Bank Construction and Resource Utilization Hefei Anhui China

**Keywords:** hybrid epithelial‐mesenchymal transition, IL6/JAK/STAT3 axis, monocyte chemotactic protein‐inducible protein‐1, pancreatic cancer, tumor stemness

## Abstract

**Introduction:**

Pancreatic cancer (PC) is a common malignant tumor with high morbidity and mortality and a very poor prognosis, highlighting the urgent need to identify molecular therapeutic targets. Monocyte chemotactic protein‐inducible protein‐1 (MCPIP1) is a common inflammatory protein associated with the pathogenesis of a variety of cancers, although a comprehensive understanding of its function and the underlying mechanisms involved in PC remains unclear.

**Materials and Methods:**

Immunohistochemistry, western blotting, immunofluorescence, flow cytometry, Transwell, and the scratch assay were used to evaluate the functional role of MCPIP1 in PC. Human PC samples, PC cells, and tumor tissues from subcutaneous tumors of nude mice were examined for MCPIP1 and a panel of epithelial‐mesenchymal transition (EMT) ‐related indicators. In the mechanistic study, the IL6/JAK/STAT3 signaling pathway was investigated as a potential downstream pathway. IL6 activity was inhibited using the pharmacological inhibitor LMT‐28 and was applied to MCPIP1 gene‐deficient cells to assess the reversal of the malignant phenotype.

**Results:**

MCPIP1 was significantly downregulated in PC tissues and its expression strongly correlated with patient survival. MCPIP1 knockdown enhanced tumor cell stemness, proliferation, migration, and hybrid epithelial‐mesenchymal transition (hybrid EMT) in PC cell lines, whereas overexpression suppressed these phenotypes. In xenograft models, MCPIP1 knockdown promoted tumor growth and hybrid EMT progression in mice. MCPIP1 knockdown activated the IL6/JAK/STAT3 signaling pathway, which was inhibited by MCPIP1 overexpression. LMT‐28 treatment reversed the stemness and hybrid EMT phenotypes of MCPIP1‐deficient cells.

**Conclusion:**

MCPIP1 is a key regulator of PC progression, acting as a tumor suppressor by inhibiting the IL6/JAK/STAT3 signaling pathway. The findings suggest that MCPIP1 is a promising therapeutic target with potential implications for the development of new strategies for managing PC.

## Graphical abstract

MCPIP1 deficiency activates the IL6‐JAK2‐STAT3 signaling pathway, driving hybrid EMT stemness and promoting pancreatic cancer proliferation, invasion, and migration.

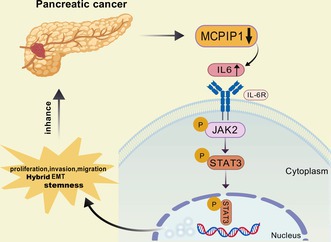

## 1 Introduction

Pancreatic cancer (PC) is the most malignant of the intestinal tract tumors, with an overall 5‐year relative survival rate of approximately 12.8% [1]. It is characterized by difficult detection and poor prognosis. The global incidence of PC has increased at an alarming rate over the past few decades. Current projections indicate that it will become the foremost cause of cancer‐related deaths worldwide [2]. Among the many pancreatic tumor types, pancreatic ductal adenocarcinoma (PDAC) is the most prevalent. In addition, a small number of patients with PC may be diagnosed with less common PC types such as adenoalveolar cell carcinomas and neuroendocrine tumors [3]. To date, most PC treatment options are ineffective, and the only treatment with a high cure rate is surgical resection [4]. However, it should be noted that surgery is only employed in a limited number of patients with non‐metastatic resectable tumors. Because PC rarely presents obvious clinical symptoms in its early stages of development and lacks early diagnostic biomarkers, approximately 50% of patients exhibit local infiltration or distant metastasis at the time of diagnosis [5], forming metastatic foci firstly in the abdomen and liver, and then in the lungs, brain, and bones [6], at which point they are no longer amenable to surgery. Consequently, there is an urgent need to explore the main molecules and mechanisms involved in PC progression.

Epithelial mesenchymal transition (EMT) is associated with wound healing, tissue fibrosis, tumor progression, and allows tumor cells to spread to new areas and invade surrounding tissues, making it one of the main drivers of cancer progression [7]. EMT is pivotal for the progression of malignant tumors, through enhanced mobility and invasive spread of cancer cells to distant organs, which in turn leads to cancer metastasis [8]. Hybrid EMT is a specific state of the EMT process that refers to cells exhibiting epithelial and mesenchymal characteristics, rather than being completely transformed from epithelial to mesenchymal cells. Furthermore, the hybrid EMT phenotype is characterized by a loss of apical‐basal polarity, enhanced motility, and maintained adhesion to neighboring cells, in addition to the acquisition of mesenchymal features. This hybrid state is more plastic than the fully epithelial or mesenchymal state [9]. EMT or hybrid EMT plays an important role in cancer pathogenesis, therapeutic resistance [10], metastasis, and stemness [11], and the mixed epithelial/mesenchymal phenotype exhibits a greater risk of metastasis and with complete EMT [12] and is more likely to lead to a poor clinical prognosis of the tumor. Tumor stemness is defined as the property of tumor cells to exhibit characteristics reminiscent of stem cells, which include self‐renewal capacity, multidirectional differentiative potential, and resistance to pharmacological interventions [13]. Tumor cells with stemness are known as tumor stem cells (CSCs) and they play a significant role in tumor growth and spread [14]. Both tumor stemness and hybrid EMT are key drivers of tumor metastasis. In ovarian cancer [15] and non‐small cell lung cancer [16], tumor cells with a hybrid EMT phenotype exhibit enhanced stem cell‐like properties. Correspondingly, these stem cell‐like tumor cells further consolidate and maintain the hybrid EMT state, thereby endowing tumor cells with stronger invasive and metastatic capabilities [17, 18]. When PCs develop into advanced stages, the tumor has an inadequate supply of blood, oxygen, and nutrients, and at this point, PCs tend to look for favorable ways to survive, and metastatic and invasive processes become an important survival behavior of PCs [19].

Monocyte chemotactic protein‐inducible protein‐1 (MCPIP1) is a recently discovered class of CCCH‐type zinc finger family molecules with immunomodulatory effects. Its molecular weight is 66 kDa. In addition, according to sequence analysis, it also has a proline‐rich region, a PIN domain, and a ubiquitin‐binding domain [20]. Tumor cell apoptosis, invasion, angiogenesis, and metastasis are all dependent on MCPIP1. Furthermore, it exerts a key regulatory function in the progression of many cancers [21, 22, 23]. Based on recent studies, MCPIP1 expression is upregulated in lung cancer and in primary neuroblastoma disorders, which have been associated with a poor prognosis [24, 25]. Overexpression of MCPIP1 is also strongly related to the long‐term survival of patients with breast cancer, as seen by decreased tumor growth and proliferation [26]. Nonetheless, angiogenesis, distant metastasis, and malignant development of renal carcinoma are also strongly correlated with the expression of MCPIP1 [27]. However, it remains unclear how MCPIP1 controls EMT and whether it has any bearing on the progression of PDAC.

The IL6/JAK/STAT3 signaling axis is aberrantly activated in a wide range of tumors, and the transduction of this signaling axis promotes tumor cell invasion of surrounding tissues, resistance to drugs, and suppression of anti‐tumor immunity [28]. Mounting evidence suggests that the JAK/STAT3 signaling pathway plays a key role in tumor metastasis and EMT, with an active IL6/JAK2/STAT3 axis and stem cell‐like characteristics contributing to the poor prognosis of metastasized breast cancers [29]. Activation of the pathway similarly promotes glioma globe tumor angiogenesis, invasion, and proliferation [30]. MCPIP1 regulates the inflammatory response by physically interacting with the stem‐loop structure in the 3′ untranslated region (UTR) of the transcripts through its structural domain PIN. This interaction results in a decrease in mRNA stability, which in turn leads to degradation of mRNA transcription of pro‐inflammatory cytokines such as IL6 [31, 32]; thus, negatively regulating inflammation. In summary, we can conclude that MCPIP1 and IL6 are directly linked and that both MCPIP1 and the IL6/JAK2/STAT3 pathway play essential roles in many cancers. Hence, it is reasonable to speculate that MCPIP1 and the IL6/JAK2/STAT3 signaling pathway play equally crucial roles in PC.

Here, we first clarified the expression and survival prognosis of MCPIP1 in tumor tissues of patients with PC using clinical samples. Secondly, to elucidate whether MCPIP1 affected PC metastasis and development, we interfered with MCPIP1 expression and examined functional phenotypes associated with hybrid EMT and tumor stemness through in vivo and in vitro experiments, which clarified that MCPIP1 played a role in inhibiting the malignant behavior of PC by acting through the IL6/JAK2/STAT3 signaling axis. Consequently, we hypothesized that MCPIP1 may represent a promising therapeutic target for future PC treatment.

## 2 Materials and Methods

### 2.1 Tissue Samples

The 14 tissue specimens selected for this study derived from patients with PC who had received treatment at the First Affiliated Hospital of Anhui Medical University. PC tissues were obtained on surgical intervention and were stored for future research in liquid nitrogen. Patients were diagnosed with PC by histopathological examination based on the analysis of the resected tissues. The procurement of all tissue samples was conducted with the explicit consent of the patients and their family members. The research protocol in this paper was approved by the Biomedical Ethics Review Committee of Anhui Medical University under the ethical approval number No. 83244511.

### 2.2 Cell Lines and Cell Culture

Two cell lines were purchased from Wuhan Procell Life Sciences Co Ltd., ASPC‐1 (CL‐0027) and PANC‐1 (CL‐0184). Short Tandem Repeats assays were used to characterize each cell line. HPDE6‐C7 (HTX1979) was obtained from Otwo Biotech (Shen Zhen) Inc. HPDE6‐C7 and ASPC‐1 cells were grown in RPMI‐1640 full media (Procell, PM150110, China), whereas the PANC‐1 was grown in DMEM full medium (Hyclone; Thermo Fisher Scientific, USA), and passage was performed when the cells reached a cell confluence of approximately 80%. All cell lines were cultured in a 37°C, 5% CO_2_ incubator. When the cells were cultured to 80% density, they were rinsed three times with sterile PBS, and then the cells were digested with trypsin to collect the cells for immunoblotting, Transwell, CCK‐8, and wound healing assays. The IL6 inhibitor LMT‐28 (HY‐102084, Med‐ChemExpress, USA) was dissolved in Dimethyl Sulfoxide (DMSO) (Sigma‐Aldrich, USA). Subsequently, sh‐MCPIP1 cells were exposed to a concentration of 10 μM for six hours.

### 2.3 qRT‐PCR

Using the SPARK easy Cellular RNA Rapid Extraction Kit (# AC0205‐A), total RNA was obtained from pancreatic tumor cells according to the manufacturer's instructions. The Spark Reverse Transcription Kit (# AG0304) was used for reverse transcription. Gene expression was evaluated in a 20 μL‐volume reaction system, which contained 1 μL cDNA, 0.4 μL of each primer, 10 μL 2× SYBR Green qPCRMix, and 8.2 μL DNAse/RNAse‐free water; each assay was performed in triplicate using a 2× SYBR Green qPCRMix with ROX (SparkJade # AH0104). PCR experiments were performed in accordance with the manufacturer's guidelines. The primer sequences are listed in Table 1.

**TABLE 1:** Primer sequence for real‐time PCR.

Gene	Primer	Sequence (5′‐3′)
MCPIP1	Forward	GGCAGTGAACTGGTTTCTGGA
	Reverse	GATCCCGTCAGACTCGTAGG
N‐cadherin	Forward	CAGACATGGAAGGCAATCCCACA
	Reverse	CTGGATGGCGAACCGTCCAGTAGGA
E‐cadherin	Forward	ACAGCCCCGCCTTATGATT
	Reverse	TCGGAACCGCTTCCTTCA
Vimentin	Forward	GCTGAATGACCGCTTCGCCAACT
	Reverse	GCTCCCGCATCTCCTCCTCGTA
β‐actin	Forward	CATGTACGTTGCTATCCAGGC
	Reverse	CTCCTTAATGTCACGCACGAT
CD44	Forward	AGAGGCTGAGACAGGAGGTT
	Reverse	GCTTCCAGAGTTACGCCTT
Nanog	Forward	TCTGGACACTGGCTGAATCC
	Reverse	TGACTGGATGGGCATCATGG
Oct4	Forward	AGGTATTCAGCCAAACGACCA
	Reverse	GCACGAGGGTTTCTGCTTTG
Sox2	Forward	TACAGCATGATGCAGGACCA
	Reverse	CGAGCTGGTCATGGAGTTGTA
Lgr5	Forward	TCTCGGTGAGCCTGAGAAAGC
	Reverse	ATGCTGGAGCTGGTAAAGGT
ALDH	Forward	TTTGTCCAGCCCACAGTGTT
	Reverse	ACGCCATAGCAATTCACCCA

### 2.4 Western Blotting

Samples were collected and then mixed with lysis buffer that contained pre‐cooled RIPA (# P0013B Beyotime) and PMSF (# ST507‐10 mL Beyotime). After ultrasonic fragmentation and ultracentrifugation, a spectrophotometer was utilized to measure the protein content of the protein supernatant. Before being transferred to Nitrocellulose membranes, protein samples were separated using 10% and 8% sodium dodecyl sulfate‐polyacrylamide gel electrophoresis. Following membrane transfer, NC membranes were blocked for two hours at room temperature using 5% BSA, and then they were exposed for the entire night at 4°C to the indicated antibodies, including MCPIP1 (# 68616‐1‐Ig, Proteintech), e‐cadherin (# 3195S, USA, CST), n‐cadherin (# 13116S, USA, CST), vimentin (# 5741S, USA, CST), IL6 (# D220828, Sangon Biotech), JAK2 (# R24775, Zen Bioscience), phosphorylated‐JAK2 (# 3776T, USA, CST), STAT3 (# R22785 Zen BioScience), phosphorylated‐STAT3 (# 9145T, USA, CST), CD44 (# 60224‐1‐Ig, Proteintech), CD133 (# 18495‐1‐AP, Proteintech), GAPDH (# R24402, Zen BioScience) and β‐actin (# D110001‐0100, Sangon Biotech). Subsequently, the matching secondary antibody was incubated for one hour at room temperature. With the help of enhanced chemiluminescence reagent (Sparkjade, #ED0015‐C), images were captured using a chemiluminescent imaging system (Tanon, China) and finally analyzed with Image J analysis software.

### 2.5 CCK‐8 Assay

Pancreatic tumor cells were digested using trypsin, counted, and inoculated in 96 well cell culture plates. The 3000 cells per well were to be standardized. At the corresponding time points of the assay, cells were removed from the incubator, 10 μL of CCK‐8 reagent (Spark Jade, CT001‐B) was added to each well, and allowed to react 1 to 4 h away from light. The OD value at 450 nm was detected on an enzyme meter (Molecular Devices Microplate Reader, SpectraMax iD3) and the linearity was generally optimal when the OD value was in the range of 0.8 to 1.5.

### 2.6 Nude Mouse Tumor Formation Assay

Twenty BALB/c male naked mice were acquired from GemPharmatech Co. Ltd. (Nanjing), and housed at the Anhui Medical University Laboratory Animal Center under specific pathogen‐free conditions. Nude mice were divided into control and knockdown groups and were housed in different cage boxes, with 5 nude mice in each box. The cells were harvested when they had grown to about 80% and cell suspensions were prepared by mixing pre‐cooled PBS and matrix gel at a ratio of 1:1 on ice. Within an hour, the cell mixture was injected into the right axilla of naked mice. Body weights of nude mice were measured every 8 days after subcutaneous injection, and tumor volumes were calculated once the tumors had started to grow. The mice were euthanized when the tumor volume reached an appropriate size, and the tumors were excised and preserved in liquid nitrogen for later use. All animal experiments in this study were approved by the Laboratory Animal Ethics Committee of Anhui Medical University (No. 20241477).

### 2.7 Lentivirus Construction and Utilization

Lentivirus overexpressing MCPIP1, vector control, shRNA (ZC3H12A) and shRNA (scramble) were purchased from BrainVTA, China. shRNAs were designed with the following target sequences: shRNA (scramble), 5′‐CCTAAGGTTAAGTCGCCCTCG‐3′, shRNA (ZC3H12A), 5′‐GCCAGCGTGTATACTAAGCTG‐3′. The TetON system lentiviral vector was used as the overexpression lentivirus in order to achieve stable MCPIP1 expression. The system relies on the LV‐tre tight‐ZC3H12A‐P2A‐EGFP‐PGK‐rTTA‐P2A‐puro‐WPRE vector. PC cell transfections were performed according to the manufacturer's instructions. In order to achieve stable overexpression of MCPIP1 in PANC‐1 and ASPC‐1, Dox (0.6 μg/mL) was added during viral infection and subsequent culture. After screening infected cells for approximately a week with puromycin (0.5 μg/mL), qRT‐PCR and immunoblotting were used to evaluate the efficacy of target gene silencing and overexpression.

### 2.8 Transwell Assay

Penetrable cell culture chambers (8.0 μm, LABSELECT) were used to evaluate the ability of cells to migrate. A total of 4 × 10^4^ PANC‐1 and ASPC‐1 cells was seeded in the upper compartment, and 10% FBS complete media was added to the lower chamber. The only difference between the migration test and the invasion test is that the upper chamber of the invasion test is pre‐coated with matrix gel (Corning, 356234). After 48 h of incubation for the migration or invasion assay, cells were fixed by adding 4% Paraformaldehyde (PFA) to the chamber for 10 min, and chambers were washed with PBS before the addition of crystal violet for approximately 20 min. After washing off the excess crystal violet with PBS, the remaining cells in the upper chamber were removed with a cotton swab. Finally, cells were observed and photographed using an inverted microscope (YUESHI, YIB510, China) at a magnification of ×100, and three randomly selected fields of view were used for cell counting.

### 2.9 Cell Scratching Assay

The cells were seeded in six‐well plates at approximately 5 × 10^5^ cells per well and were allowed to grow until fully confluent. Subsequently, scratches were made vertically in the cell dish using pipette gun tips. The scratched cells were washed with PBS, and the culture medium containing 1% FBS was added to the well plates to continue incubation for 48 h. Pictures were taken using a microscope (YUESHI, YIB510, China) with different fields of view at 0 and 48 h, respectively.

### 2.10 Immunofluorescence

A cell crawler was placed in a 12‐well plate; trypsin digestion was performed to collect cells and seed them on the crawls. Subsequently, once the cell growth reached approximately 50%, cells were fixed with 4% PFA for 15 min at room temperature, and the remaining PFA was washed with PBS; the cells were then blocked with Immunostaining Blocking Solution (# P0260, Beyotime) for 15 min. Finally, primary antibodies (including N‐cadherin, E‐cadherin, and vimentin) were added and the culture continued overnight at 4°C in a refrigerator. After the primary antibody incubation was completed, excess antibody was washed off with PBS before incubating with Alexa Fluor594 (Invitrogen, # A‐11012) for 1 h at room temperature away from light. Finally, the plate was sealed with a droplet of anti‐fluorescence quencher containing DAPI and fixed with nail polish. Finally, pictures were taken using a fluorescence microscope with a magnification of ×200.

### 2.11 Immunohistochemistry

After dehydrating and embedding the tissue in paraffin, the tissue was cut into 5 μm slices. After baking the paraffin slices, they were dewaxed in xylene and 100%/90%/80%/70% alcohol gradient in sequence. Afterwards, the slices were placed in a box containing a citrate solution of EDTA, and then the slices were placed in a water bath for 30 min at 100°C. At the end of the 30 min, the slices were removed and cooled to indoor temperature. The entire procedure was performed using the Universal 2‐Step Assay Kit (# PV‐9000, ZSGB‐BIO), and the rest of the procedure was performed according to the manufacturer's instructions. Anti‐MCPIP1 (#68616‐1‐Ig, Proteintech), N‐cadherin (#13116S, CST, USA), and E‐cadherin (#3195S, CST, USA) were used; vimentin (#5741S, CST, USA) and phosphorylated‐STAT3 (#9145T, CST, USA) were used as the primary antibodies. After incubation of the secondary antibody, color development was performed using the DAB Colorimetric Kit (# ZLI‐9018, ZSGB‐BIO). After rinsing with running water, nuclei were marked with hematoxylin (#C0107‐100 mL, Beyotime). Nuclei were marked with hematoxylin (#C0107‐100 mL, Beyotime) following a rinse under running water, differentiated with Ethanol Hydrochloride Rapid Differentiation Solution (#C0165S, Beyotime), and stained with Hematoxylin Re‐blueing Solution (#G1040‐500ML, Servicebio). After rinsing with running water, the sections were sequentially dehydrated in 80%/90%/100% alcohol, xylene, and sealed with gum. The staining results were observed under a microscope and photographed at a magnification of ×200.

### 2.12 Tumor Sphere Formation Assay

The cells were digested into a single cell suspension to ensure that the cells were evenly dispersed, seeded into ultra‐low adsorption six‐well plates (Corning, USA) at a density of approximately 1 × 10^4^ cells per well, and cultured in a specialized medium for sphere formation consisting of serum‐free DMEM/F‐12 (MedChemExpress) supplemented with β‐FGF (10 ng/mL, MedChemExpress), B27 (2%, MedChemExpress), human EGF (20 ng/mL, MedChemExpress), and IGF (20 ng/mL, MedChemExpress). Cells were then cultured at 37°C in an atmosphere containing 5% CO_2_ to form tumor spheres. After 10 to 14 days, tumor sphere formation was observed by inverted microscopy, and cell images were captured at a magnification of ×100. The number of tumor spheres was meticulously counted and graphically represented. The percentage of tumor spheres exhibiting diameters of 50 to 100 μm, 100 to 150 μm, or > 150 μm was calculated and graphically represented using ImageJ software.

### 2.13 Flow Cytometry

Cell cycle of cells under different treatments was detected using the Cell Cycle Assay Kit (#BB‐4104, Bestbio) following the kit instructions. The results were detected by Flow Cytometer (BD FACSCelesta). The excitation wavelength was 488 nm. The analysis of cellular DNA contents and light scattering was conducted using FlowJo version 10.8.1.

### 2.14 Database

The Kaplan–Meier (https://kmplot.com/analysis) is capable of evaluating the correlation between the expression of all genes and survival rates in multiple tumor types. This online tool was used to analyze the correlation between the overall survival (OS) of pancreatic cancer (*n* = 99) and MCPIP1. The Log‐rank test was employed for comparison, yielding a P value of 0.0339 (accessed in March 2023). The protein–protein interaction network was constructed using the STRING database (https://cn.string‐db.org/), with a medium confidence level (comprehensive score ≥ 0.7) for the interaction network (accessed in June 2024).

### 2.15 Statistics

The experiments in this paper were repeated at least in triplicate. Data were expressed as mean ± standard deviation (SD) using GraphPad Prism v.9.0 as the statistical tool. Data was statistically analyzed using Student's t‐test and One‐Way ANOVA.

## 3 Results

### 3.1 Low Expression of MCPIP1 Was Strongly Associated With a Poor Prognosis

**FIGURE 1:**
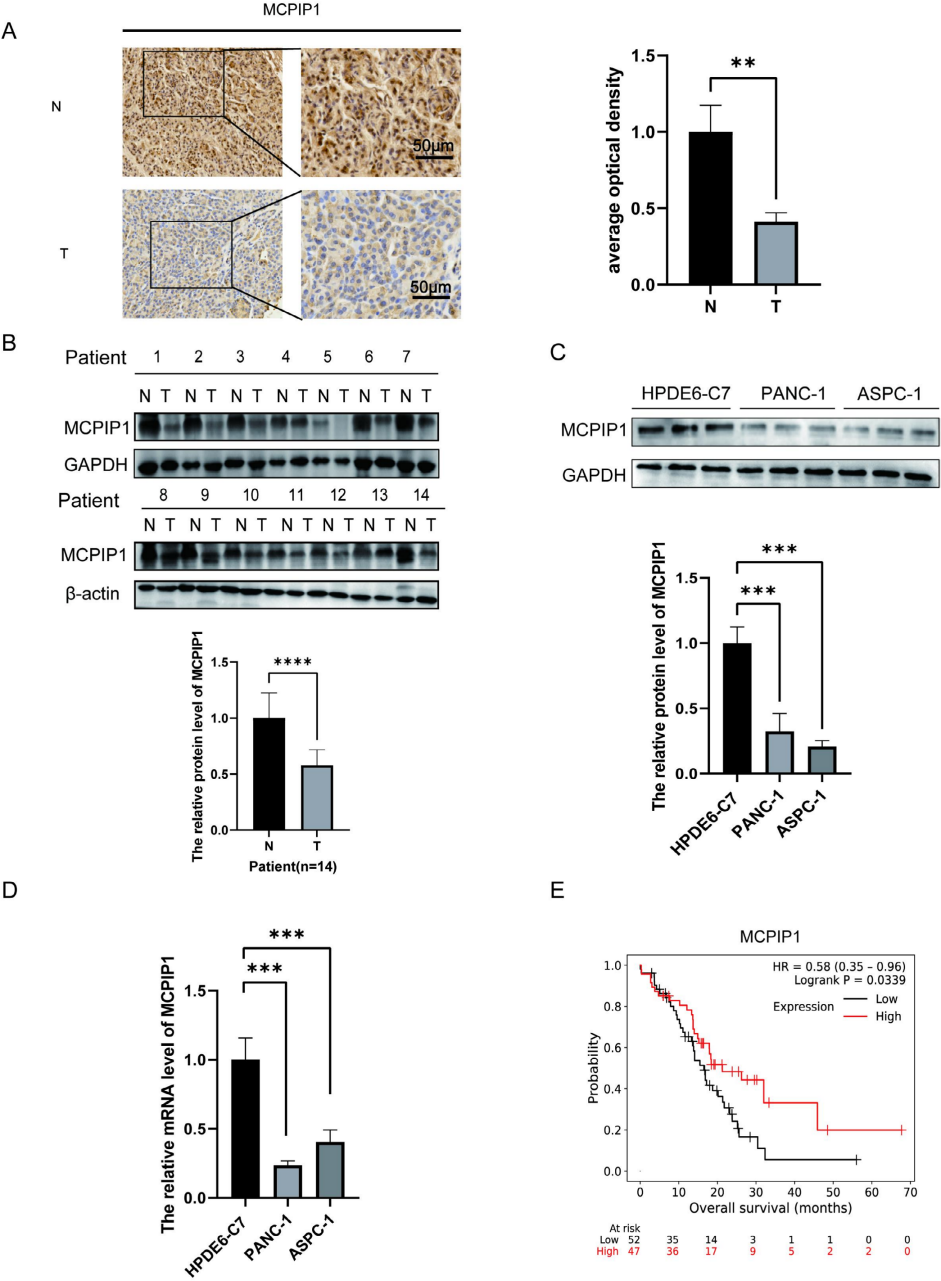
Low MCPIP1 expression is strongly correlated with a poor prognosis. (A) Immunohistochemical analysis of MCPIP1 in normal pancreatic tissue (N) and pancreatic tumor (T) showing adenocarcinoma tissue that is a moderate to poorly differentiated (magnification ×200; scale bar 50 μm). (B) Western blot analysis revealed the presence of MCPIP1 expression in pancreatic tumor tissues. (C) Expression of MCPIP1 in normal cells of HPDE6‐C7, ASPC‐1, PANC‐1 determined by western blotting. (D) MCPIP1 mRNA levels in normal cells of human pancreatic ductal epithelium cells and pancreatic tumor cells were analyzed with qRT‐PCR. (E) Survival curves for the overall survival of pancreatic cancer using the Kaplan–Meier plotter database (*n* = 99). The mean ± SD of the three experimental groups. Statistical significance of comparisons is indicated by **p < 0.01, ***p < 0.001, and ****p < 0.0001.

To examine the function of MCPIP1 in PDAC, we used immunohistochemistry and immunoblotting analysis to determine the amount of MCPIP1 in tumor tissues from patient samples. Unlike the tumor tissues, we found that normal pancreatic tissues had higher levels of MCPIP1 (Figure 1A,B). Furthermore, MCPIP1 expression was higher in normal cells of the human pancreatic ductal epithelium than in pancreatic tumor cells, according to qRT‐PCR and western blotting findings (Figure 1C,D). Furthermore, MCPIP1 levels and patient survival had a positive correlation according to the Kaplan–Meier dataset (Figure 1E). Furthermore, MCPIP1 levels were reduced in tumor samples and were positively associated with patient survival.

### 3.2 Knockdown of MCPIP1 Promotes the Growth, Invasion, and Motility of Pancreatic Tumor Cells and Enhances Hybrid EMT

**FIGURE 2:**
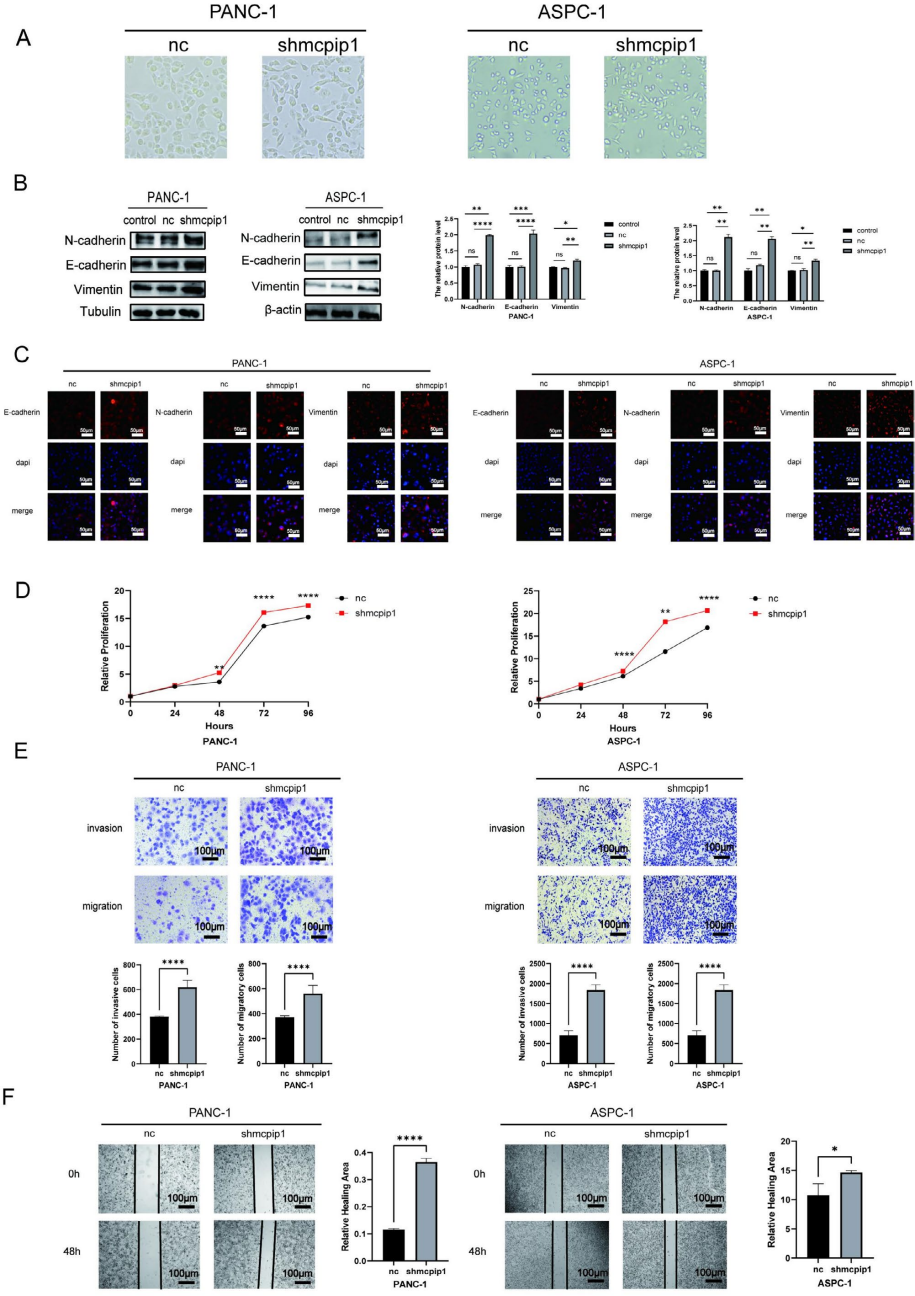
Knockdown of MCPIP1 promotes growth, encroachment, and motility of pancreatic tumor cells and improves Hybrid EMT. (A) Changes in cell morphology after knockdown of MCPIP1 in PANC‐1 and ASPC‐1. (B) Changes in the E‐cadherin, N‐cadherin, and vimentin protein levels after down‐regulation of MCPIP1 in tumor cells as shown by immunoblotting. (C) E‐cadherin, N‐cadherin, and vimentin expression levels were measured with immunofluorescence (magnification ×20; scale bar 50 μm for the two cell lines mentioned above). NC, negative control, shMCPIP1. short hairpin RNA targeting MCPIP1. (D) The proliferation rate of PANC‐1 and ASPC‐1 cells was determined using the CCK‐8 assay. (E) Cell invasion and migration were assessed by a Transwell assay, and the results were compared between the knockdown group and the negative control group, as well as statistical graphs (scale bar 100 μm; magnification ×100). (F) The level of wound healing of the tumor cell negative control and knockdown group was determined by a cell scratch assay and statistical plots of wound healing capacity and relative healing area (scale bar 100 μm; magnification ×100). Control, no treatment, NC; negative control; shMCPIP1, short hairpin RNA targeting MCPIP1. The mean ± SD of the three experimental groups is shown. Statistical significance of comparisons is defined by *p < 0.05, **p < 0.01, ***p < 0.001, and ****p < 0.0001.

EMT is widely recognized as a key process that drives tumor progression. In this paper, we investigated the effects of knocking down the expression of MCPIP1 on EMT‐related proteins in tumor cell lines. We first silenced MCPIP1 expression in pancreatic tumor cells (Figure S1A,B). During cell culture, the morphology of the knockdown cell lines PANC‐1 and ASPC‐1 changed from regular cuboidal or elliptical to spindle‐shaped or stellate (Figure 2A). Additionally, western blotting and qRT‐PCR results showed that silencing of MCPIP1 significantly increased the levels of E‐cadherin, N‐cadherin, and vimentin proteins (Figures 2B and S1C), which were consistent with immunofluorescence results (Figure 2C). We also investigated how PC cell growth, invasion, and motility were affected by MCPIP1 silencing. Using the CCK‐8 assay, we found that pancreatic tumor cells with MCPIP1 knockdown exhibited much greater absorbance (Figure 2D). This unequivocally revealed that MCPIP1 knockdown stimulated the proliferation of pancreatic tumor cells. Additionally, the effects of MCPIP1 on the migration capacity of pancreatic tumor cells were assessed using Transwell and scratch assays. MCPIP1 knockdown resulted in significantly higher levels of cell migration and invasion compared with the negative controls (Figure 2E), and the cells in which MCPIP1 expression was knocked down exhibited greater wound healing, according to the cell scratch assay (Figure 2F). Our findings implied that MCPIP1 downregulation stimulated the growth, migration, and invasion of tumor cells as well as the hybrid EMT.

### 3.3 Overexpression of MCPIP1 Inhibited the Proliferation, Invasion, and Migration of PC Cells and Hybrid EMT

**FIGURE 3:**
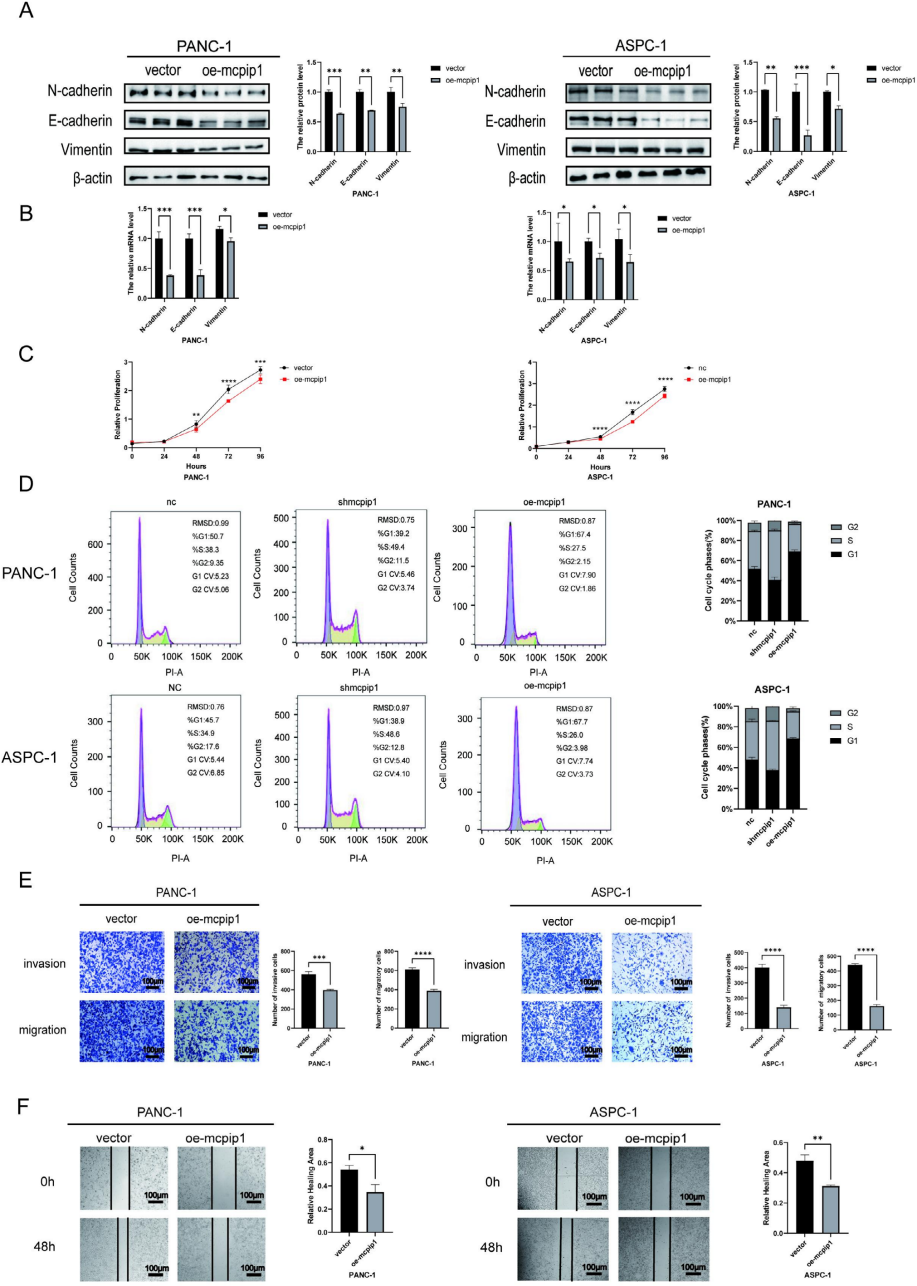
Higher levels of MCPIP1 reduced the growth, invasion, and migration of pancreatic tumor cells and attenuated EMT. (A) Changes in the E‐cadherin, N‐cadherin, and vimentin proteins after upregulation of MCPIP1 in tumor cells as analyzed by immunoblotting. (B) The expression of mRNA of E‐cadherin, N‐cadherin, and vimentin in pancreatic tumor cells as determined by qRT‐PCR. (C) The proliferation rates of the control cells and MCPIP overexpression in PANC‐1 and ASPC‐1 cells were determined using the CCK‐8 assay. (D) Cell cycle changes after overexpression of PANC‐1 and ASPC‐1 after MC knockdown, respectively. (E) Quantification and statistical analysis of cell invasion and migration capabilities from the MCPIP1 overexpression group and the negative control group derived from the Transwell assay, respectively (scale bar, 100 μm). (F) Wound healing of the tumor cell negative control and MCPIP1 overexpression group was determined by a cell scratch assay and relative statistical analysis of wound healing capacity and relative healing area (scale bar, 100 μm). NC, negative control; shMCPIP1, short hairpin RNA targeting MCPIP1; oe‐MCPIP1, overexpression of MCPIP1; vector, empty vector lentivirus; and oe‐MCPIP1, overexpression of MCPIP1. The mean ± SD of the three experimental groups. Statistical significance of comparisons is indicated by *p < 0.05, **p < 0.01, ***p < 0.001 and ****p < 0.0001.

Next, we overexpressed MCPIP1 in pancreatic tumor cell lines to further validate its function (Figure S1D,E). Immunoblotting results showed that MCPIP1 overexpression greatly reduced the level of proteins E‐cadherin and N‐cadherin (Figure 3A). qRT‐PCR further validated the decreased expression of E‐cadherin, N‐cadherin, and vimentin proteins after MCPIP1 overexpression in tumor cells (Figure 3B). We then investigated how MCPIP1 overexpression affected the proliferation, invasion, and motility of pancreatic tumor cells. The CCK‐8 assay revealed that pancreatic tumor cells overexpressing MCPIP1 had considerably reduced absorbance (Figure 3C). This clearly indicated that MCPIP1 overexpression hindered PC cell proliferation. In addition, we examined changes in the cell cycle of PC cells following MCPIP1 overexpression by flow cytometry, and the results showed that the S‐phase and G2‐phase of the cell cycle in the knockdown group were significantly increased, whereas in the overexpression group, the S1‐phase of the cells was enhanced (Figure 3D). The association between MCPIP1 and the capacity of pancreatic tumor cells to migrate was further evaluated using Transwell and scratch assays. Cell lines that overexpressed MCPIP1 had significantly reduced cell migration and invasion (Figure 3E), whereas the cell scratch assay revealed that the ability of MCPIP1‐overexpressing PANC‐1 and ASPC‐1 cells to heal wounds was also compromised (Figure 3F). Our findings revealed that MCPIP1 overexpression impeded EMT, as well as the migration, invasion, and proliferation of pancreatic tumor cells.

### 3.4 MCPIP1 Regulates the Stem Cell Characteristics and Resistance to Gemcitabine in PC Cells

**FIGURE 4:**
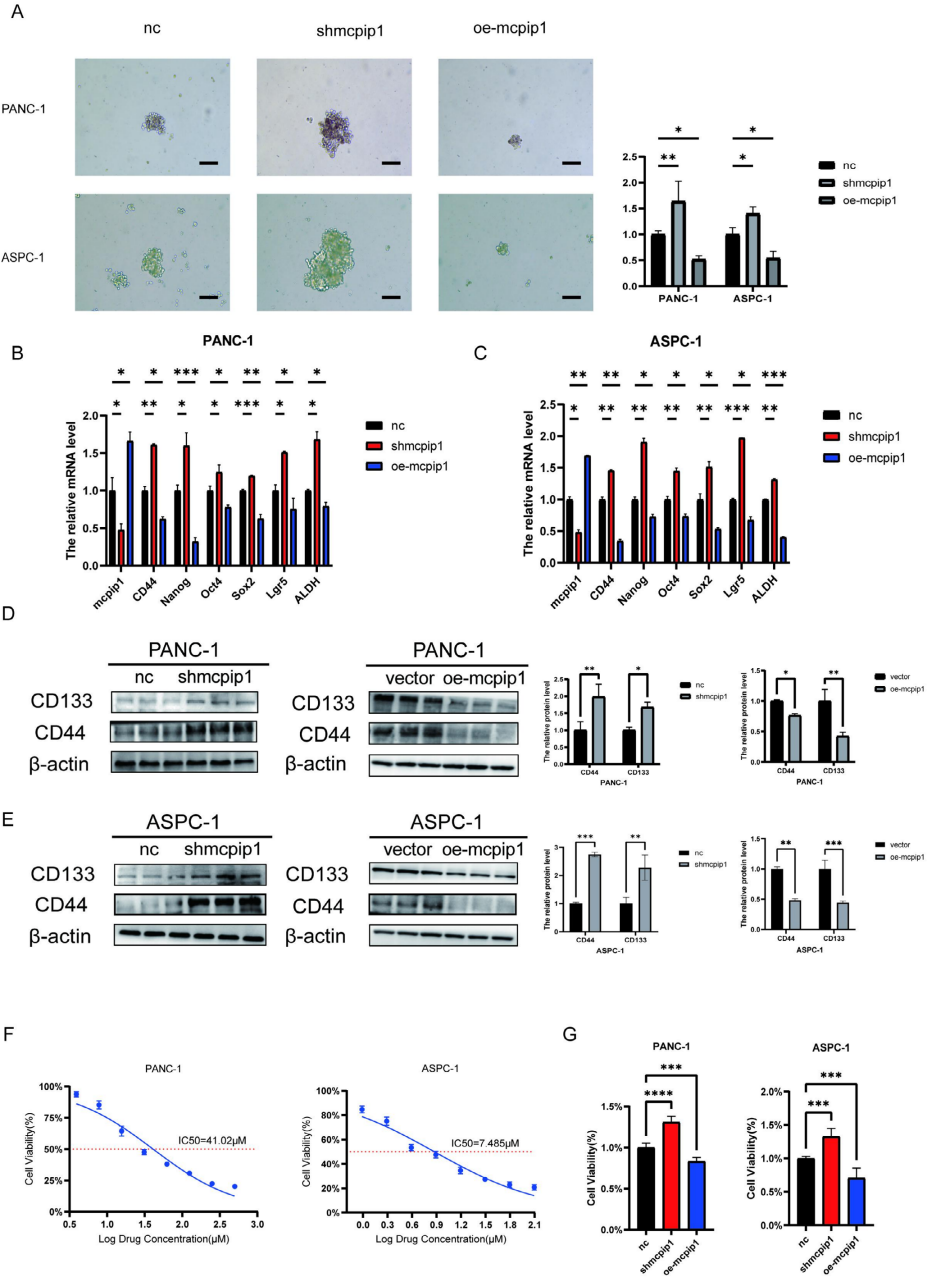
MCPIP1 regulates the stemness characteristics and resistance to gemcitabine in pancreatic cancer cells. (A) Differences in sphere formation were detected using the tumor sphere formation assay after the knockdown of PANC‐1 and ASPC‐1 expression and overexpression of MCPIP1 (magnification ×100; scale bar 100 μm). (B) The qRT‐PCR results showed that knockdown and overexpression of MCPIP1 in the PANC‐1 cell line resulted in decreased and increased expression of tumor stemness markers, respectively. (C) The qRT‐PCR results showed that knockdown and overexpression of MCPIP1 in the ASPC‐1 cell line resulted in decreased and increased expression of tumor stemness markers, respectively. (D) Western blot detection of changes in stemness markers CD44 and CD133 after intervention with MCPIP1 in the PANC‐1 cell line. (E) Western blot detection of changes in stemness markers CD44 and CD133 after intervention with MCPIP1 in the ASPC‐1 cell line. (F) Gemcitabine IC50 measured by CCK‐8 in PANC‐1 and ASPC‐1 cells. (G) Cells from control, knockdown and overexpression groups were treated simultaneously with this gemcitabine IC50, and the CCK‐8 assay showed that pancreatic cancer cells with oe‐MCPIP1 showed survival inhibition. NC, negative control group, shMCPIP1, short hairpin RNA targeting MCPIP1; vector, empty vector lentivirus, oe‐MCPIP1, overexpression of MCPIP1. The mean ± SD of the three experimental groups. Statistical significance of comparisons is indicated by *p < 0.05, **p < 0.01, ***p < 0.001 and ****p < 0.0001.

The concept of cancer stemness has emerged as a pivotal potential mechanism contributing to the development and progress of pancreatic cancer. To determine how MCPIP1 is associated with the stem‐like properties of PC cells, we evaluated how quickly tumor cells could grow into spheres. We found that when MCPIP1 gene expression was suppressed, the cells formed more spheres. However, when MCPIP1 was overexpressed, the cells formed fewer spheres (Figure 4A). Similarly, by using qRT‐PCR and western blotting, we found that knockdown of MCPIP1 considerably enhanced the level of stemness markers including CD133 and CD44, whereas MCPIP1 overexpression reduced the expression of CD133 and CD44 (Figure 4B–E). Furthermore, to assess the potential role of MCPIP1 in cellular resistance to chemotherapy, we treated PC cells with gemcitabine (Gem), a first‐line therapeutic agent for PC. We first determined the IC50 of Gem for two untransfected cell lines, PANC‐1 and ASPC‐1 (Figure 4F). The cells of the control, MCPIP1 knockdown, and MCPIP1 overexpression groups were then simultaneously treated with this IC50 for 72 h. The CCK‐8 assay showed that the viability of sh‐MCPIP1 transfected cells was significantly higher than that of control cells, whereas the viability of transfected cells in the overexpression group was lower than that of the control group (Figure 4G). These data suggest that MCPIP1 can regulate stem cell permeability and Gem resistance in PC cells.

### 3.5 Knock‐Down of MCPIP1 Promoted Tumorigenicity in Mice In Vivo

**FIGURE 5:**
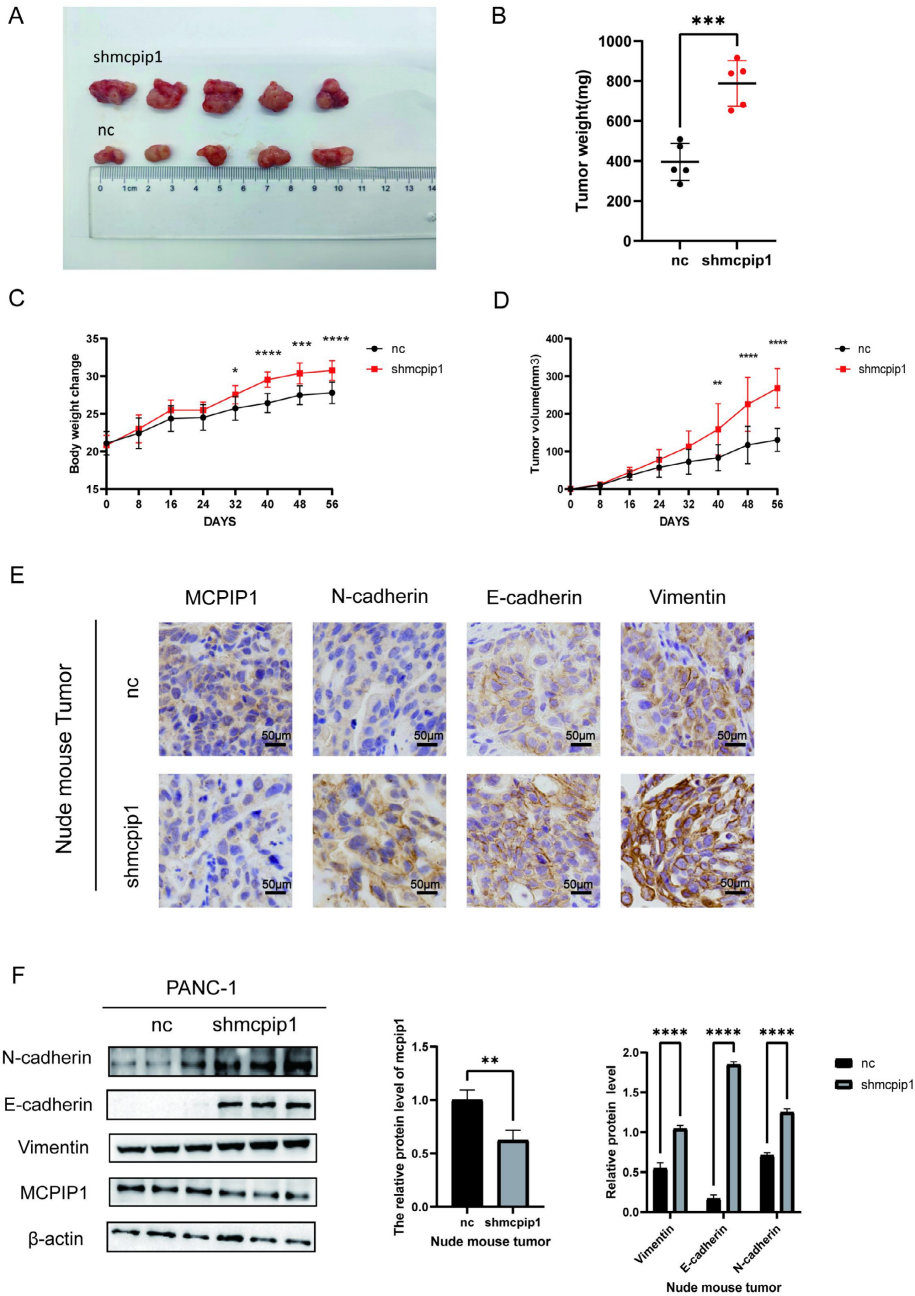
MCPIP1 knockdown stimulates the development of pancreatic tumors and EMT in nude mice. (A) Fifty‐six days after the subcutaneous injection of tumor cells, the size of the subcutaneous tumors was measured in experimental and control nude mice. Each group consisted of five mice. (B) Statistical graph of the weights of two groups after tumor excision in mice. (C, D) Statistical graph of changes in weight and tumor size during development in mice. (E) IHC staining of the PANC‐1‐derived tumor control and knockdown groups for MCPIP1, E‐cadherin, N‐cadherin, and vimentin (magnification ×200; scale bar 50 μm). (F) Immunoblotting showing the expression of MCPIP1, E‐cadherin, N‐cadherin, and vimentin in PANC‐1‐derived tumor tissues. NC, negative control; shMCPIP1, short hairpin RNA targeting MCPIP1. The mean ± SD of the three experimental groups. Statistical significance of comparisons is indicated by *p < 0.05, **p < 0.01, ***p < 0.001 and ****p < 0.0001.

An animal model was used to examine how MCPIP1 influenced tumor growth and EMT. In nude mice, the shMCPIP1 group achieved a significant decrease in tumor weight and volume (Figure 5B–D). MCPIP1 levels were lower in the tumor tissues of the MCPIP knockdown group, whereas vimentin, N‐cadherin, and E‐cadherin levels were higher than those of the control group (Figure 5E,F). Overall, silencing of MCPIP1 enhanced tumor development and hybrid EMT, according to mouse experiment results.

### 3.6 MCPIP1 Was Involved in the IL6/JAK/STAT3 Signaling Pathway in PC

**FIGURE 6:**
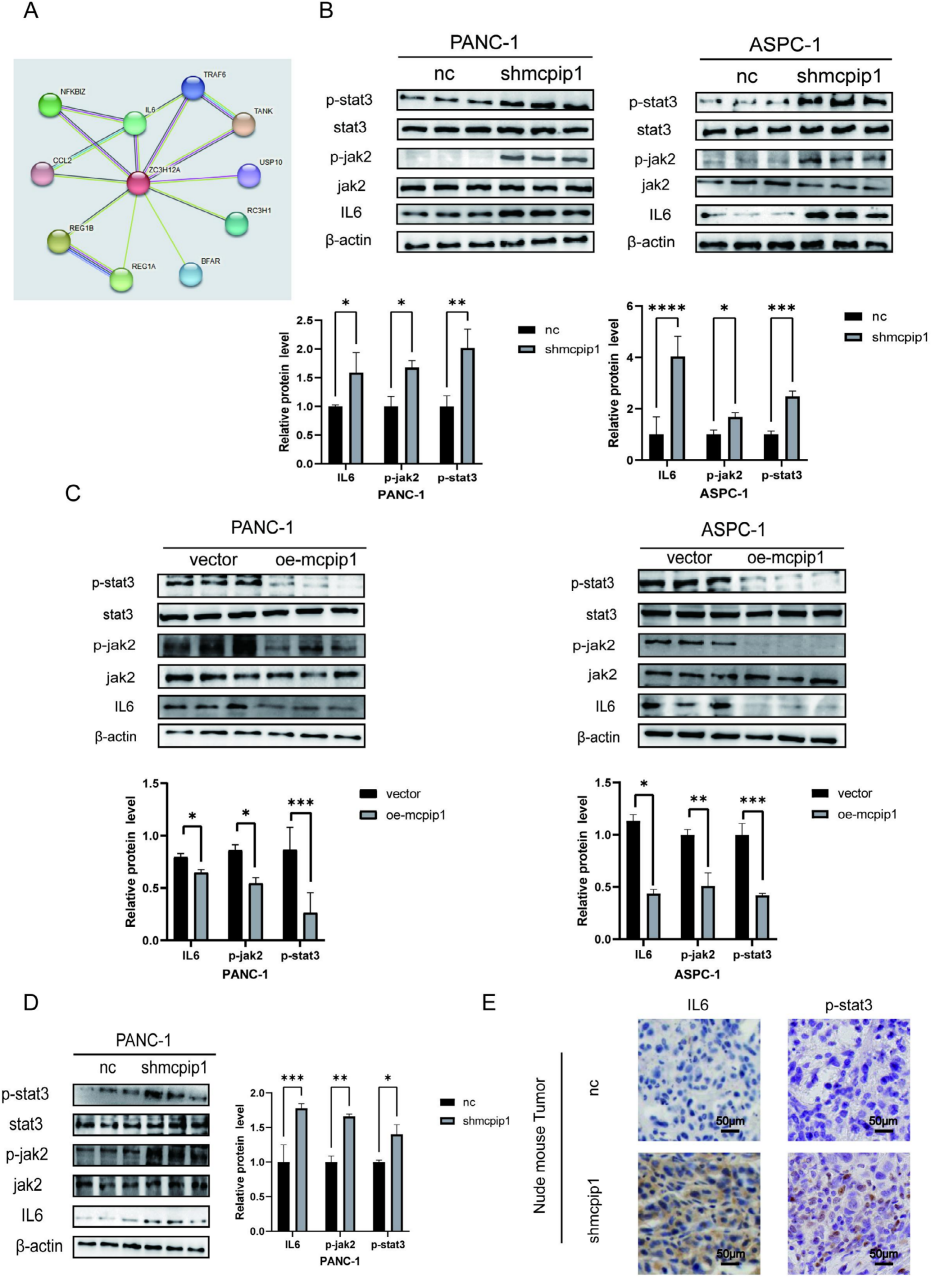
MCPIP1 is involved in the IL6/JAK/STAT3 signaling pathway in pancreatic cancer. (A) MCPIP1 protein interaction graph obtained from the STRING database. (B) After silencing MCPIP1 expression in pancreatic tumor cells, protein expression of IL6, P‐JAK2, P‐STAT3 in the above two cell lines were measured and analyzed by western blotting, and their statistical analysis is shown. (C) After overexpression of MCPIP1 in pancreatic tumor cells, protein expression of IL6, phosphorylation (P)‐JAK2, and P‐STAT3 was measured by immunoblotting; the statistical analysis is shown in graphs. (D) Detection of the levels of IL6, P‐JAK, and P‐STAT3 in the subcutaneous tumors of nude mice by immunoblotting; the statistical analysis is shown in graphs. (E) Immunohistochemical staining was used to examine IL6 and P‐STAT3 expression in subcutaneous tumors of nude mice (magnification ×200; scale bar 50 μm). NC, negative control group, shMCPIP1; short hairpin RNA targeting MCPIP1; vector, empty vector lentivirus, oe‐MCPIP1, overexpression of MCPIP1. The mean ± SD of the three experimental groups. Statistical significance of comparisons is indicated by *p < 0.05, **p < 0.01, ***p < 0.001 and ****p < 0.0001.

MCPIP1 suppresses inflammation by degrading the mRNA of pro‐inflammatory cytokines, such as IL6 [32]. Using the String database, we extracted the network diagram of MCPIP1 interaction with other proteins, where a strong interaction with IL6 could be identified (Figure 6A). In the pathogenesis of cancer, elevated levels of IL6 activated the JAK/STAT signaling pathway, which is typically linked to a poor prognosis for patients [28]. Thereby, following MCPIP1 overexpression and knockdown, we then examined changes in the IL6/JAK/STAT3 axis in pancreatic tumor cells. According to the western blotting results, MCPIP1 knockdown enhanced the expression of IL6, phosphorylated (P)‐JAK2, and P‐STAT3 proteins in ASPC‐1 and PANC‐1 cell lines, indicating that the IL6/JAK2/STAT3 signaling pathway was activated (Figure 6B). In contrast, as shown by lower levels of IL6, P‐JAK2, and P‐STAT3 proteins, overexpression of MCPIP1 led mainly to suppression of the IL6/JAK/STAT3 axis (Figure 6C). Furthermore, similar validation experiments were performed on subcutaneous tumors of naked mice. Western blotting findings confirmed that the mice in the MCPIP1 knockdown group had higher levels of P‐STAT3, P‐JAK2, and IL6 in their tumor tissues than in those of the control group (Figure 6D). Similarly, the immunohistochemical results of the knockdown group demonstrated higher positive results for IL6 and P‐STAT3 (Figure 6E). In conclusion, MCPIP1 inhibited the IL6/JAK/STAT3 axis in pancreatic tumors.

### 3.7 MCPIP1 Suppressed Hybrid EMT and Tumor Stemness Through IL6/JAK2/STAT3 Signaling

**FIGURE 7:**
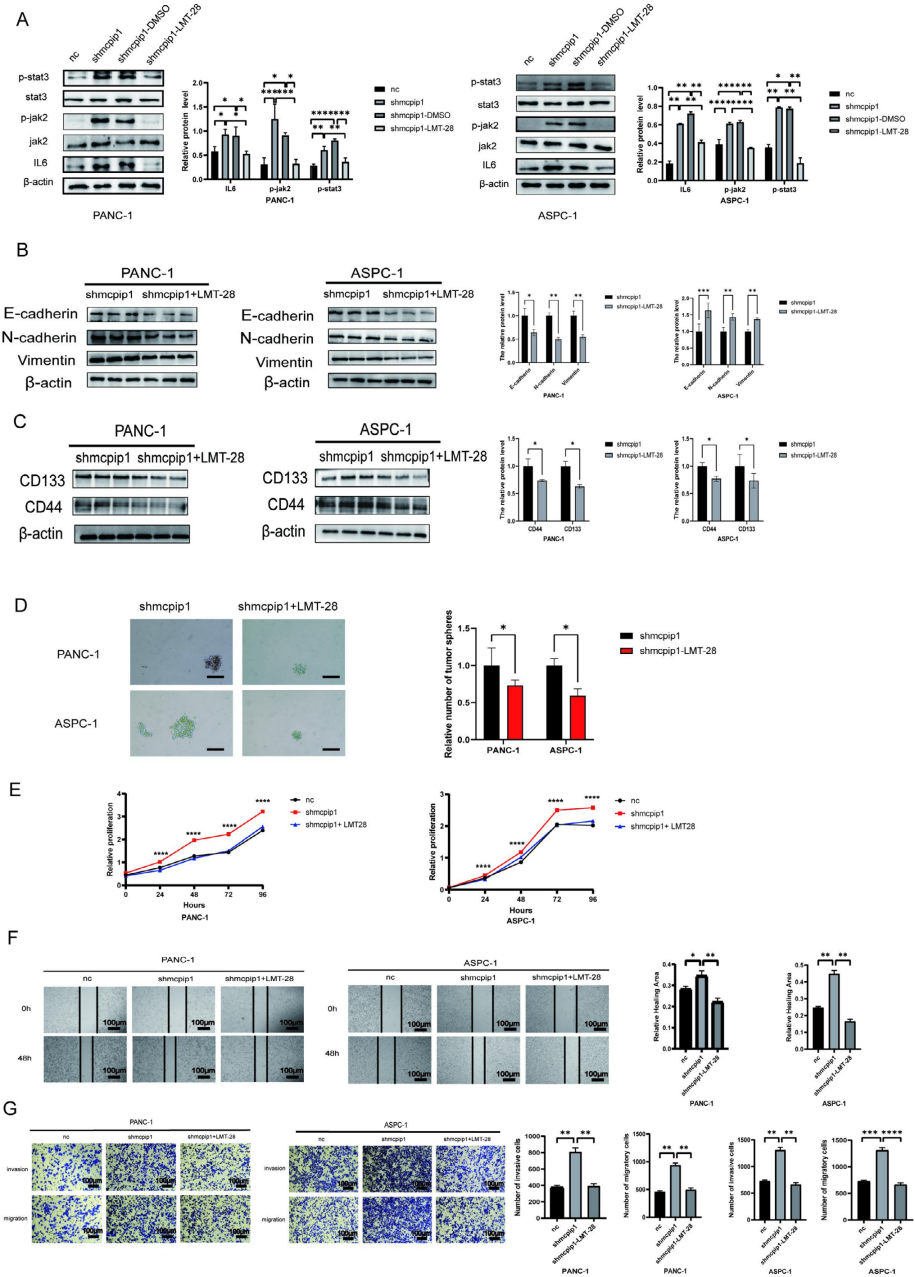
MCPIP1 inhibits mixed EMT and tumor stemness through IL6/JAK2/STAT3 signaling. (A) shMCPIP1 pancreatic tumor cell lines were exposed with 10 μM LMT‐28 for 6 h. Protein levels of IL6, phosphorylated (P)‐JAK2, and P‐STAT3 was examined by immunoblotting. (B) Western blot showing changes in EMT‐related proteins in pancreatic cancer knockdown cell lines exposed to LMT‐28. (C) Western blotting indicated changes in CD44 and CD133 proteins in pancreatic cancer knockdown cell lines exposed to LMT‐28. (D) Changes in tumor sphere formation after the addition of the IL6 inhibitor were detected (magnification ×100; scale bar 100 μm). (E) Cell Count Kit‐8 was used to identify alterations in the cell growth capacity of shMCPIP1 pancreatic tumor cells exposed to 10 μM LMT‐28. (F) Wound healing of pancreatic cancer cells from the shMCPIP1 control group was detected by a cell scratch assay in the presence of 10 μM LMT‐28 (scale, 100 μm). (G) Number of invasive cells and pancreatic cancer cell migrating cells in the control group shMCPIP1 detected by the Transwell assay in the presence of 10 μM LMT‐28 (magnification ×100; scale bar 100 μm). NC, negative control group; shMCPIP1, short hairpin RNA targeting MCPIP1; shMCPIP1 + DMSO, shMCPIP1 group treated with DMSO; and shMCPIP1 + LMT‐28, shMCPIP1 group treated with LMT‐28. The mean ± SD of the three experimental groups. Statistical significance of comparisons is indicated by *p < 0.05, **p < 0.01, ***p < 0.001 and ****p < 0.0001.

To gain further insight into whether MCPIP1 intervenes in PC hybrid EMT and tumor stemness by regulating the IL6/JAK/STAT3 signaling pathway, we treated sh‐MCPIP1 PC cell lines with the IL6 inhibitor LMT‐28. LMT‐28, a derivative of oxazolidinone, exerts a substantial inhibitory effect on IL6 activity through a direct interaction with GP130 [33]. Western blotting results indicated that the protein levels of IL6, P‐JAK2, and P‐STAT3 were considerably decreased in the two shMCPIP1 PC cell lines treated with LMT‐28 (Figure 7A), suggesting that the IL6 inhibitor had inhibited the activation of the pathway. Western blotting showed that the levels of EMT‐related proteins were reduced in both PC cell lines after the addition of LMT‐28 (Figure 7B), and the expression of the tumor stem markers CD44 and CD133 was similarly reduced (Figure 7C). The results of the tumor sphere formation assay were also in agreement (Figure 7D). Next, using the CCK‐8, we found that PC cell lines in the sh‐MCPIP1 group treated with LMT‐28 had significantly lower proliferative capacity (Figure 7E). These findings suggest that LMT‐28 attenuated the proliferative capacity of sh‐MCPIP1 PC cells by inhibiting this pathway. Similarly, the LMT‐28‐treated shMCPIP1 PC cell line showed significantly reduced migration and invasion ability and decreased wound healing rate, as detected by Transwell and the wound healing assays (Figure 6F,G). The results of the tumor spheroid formation assay indicated that the stemness of PC cells was reduced by adding the inhibitor. Thus, our results suggested that MCPIP1 reduced hybrid EMT and tumor stemness in PC cells by inhibiting the IL6/JAK2/STAT3 signaling axis.

## 4 Discussion

Despite efforts by researchers in recent years to develop new therapeutic strategies, including improved surgical approaches and adjuvant therapeutic regimens such as new chemotherapy and radiotherapy, and despite the significant advances in modern medicine, the outcomes for patients with pancreatic tumors remain deeply disheartening. PC was and still is the number one killer of patients with cancer, both historically and currently [34]. Therefore, identifying suitable molecular targets, formulating targeted immunotherapies, and improving our understanding of the underlying molecular mechanisms of this disease are essential for designing new therapeutic strategies for patients and successfully treating PC [35]. MCPIP1 is closely associated with tumorigenesis. MCPIP1 expression was negatively correlated with clear cell renal cell carcinoma (ccRCC) progression, tumor grade, and tumor vascular distribution, and in ccRCC cells, MCPIP1 depletion was found to significantly enhance tumor cell viability and proliferation [21]. In breast cancer, MCPIP1 levels were also negatively correlated with tumor grade, and reduced expression of MCPIP1 was associated with improved metastatic features [20]. In addition, MCPIP1 is also involved in the process of immune elimination of cancer cells. MCPIP1 inhibits polyubiquitination of death receptor 5 (DR5) through its deubiquitinating enzyme activity, which then enhances the lysosomal degradation of DR5, leading to resistance to DR5 activation or TRAIL‐induced apoptosis in cancer cells [36]. miR‐421 specifically targets and binds to the 3′‐UTR region of MCPIP1 and downregulates MCPIP1 expression in vivo and in vitro to promote osteosarcoma progression [37]. However, the effects of MCPIP1 on the biological properties of PC cells and the underlying molecular mechanisms are unknown. In this study, we found that the expression of MCPIP1 in PC tissues was significantly lower than that in non‐tumor tissues and was positively correlated with the PC patient survival time, suggesting that MCPIP1 is expected to be a molecular marker for PC metastasis and prognosis.

EMT is a complex transformation process in which epithelial cells no longer retain their cellular adhesion but gain the ability to move and migrate [38]. EMT is widely regarded as a prerequisite for tumor cells to become motile and malignant, thus leading to widespread metastasis and recurrence of numerous cancers [39]. It is worth mentioning that EMT is not a single process. Instead, it involves a series of different stages, such as epithelial mesenchymal cells, and a mixture of epithelial and mesenchymal transition states [40]. The EMT program does not merely switch between the epithelial and mesenchymal states, when cancer cells receive an EMT signal, they are rarely able to execute the full EMT program to enter the fully mesenchymal state, but instead maintain a different mixed EMT state, with cells in the mixed EMT state displaying both epithelial and mesenchymal features [41, 42, 43, 44]. In our study, in addition to the increased expression of detected mesenchymal markers, the epithelial marker E‐cadherin was similarly increased, which is consistent with the characteristics of hybrid EMT. A study by Weinberg and Blanpain et al. showed that cells with mixed epithelial‐mesenchymal characteristics are not only endowed with stronger tumor cell proliferation properties, but also had greater cellular plasticity, stemness, invasiveness, and metastasis than cells rich in epithelial or mesenchymal characteristics alone [45, 46, 47]. Tumor stemness refers to the potential ability of tumor cells to constantly self‐renew and proliferate and is a driving force for tumor recurrence and metastasis. Cells in the mixed EMT state improve migration by acquiring mesenchymal properties while maintaining proliferative capacity by retaining epithelial properties, which is highly consistent with the properties of tumor stem cells [11]. Shibue et al. noted that tumor stem cells and cells with mixed EMT status present enhanced resistance to chemotherapy and targeted therapies by activating common signaling pathways (e.g., TGF‐β, WNT/β‐catenin) [7]. Thus, targeting mixed EMT and cancer stemness has become a key direction in cancer therapy. We found that MCPIP1 knockdown promoted malignant behavior, mixed EMT, and tumor stemming in PC cells, and similarly confirmed that overexpression of MCPIP1 increased cellular sensitivity to Gem and inhibited tumor stemming in PC.

The IL6/JAK2/STAT3 pathway is key to tumorigenesis, progression, metastasis, and immune escape, and the transcription factor STAT3 controls how quickly cells grow, move, and spread in a number of different types of cancer. JAK2 is a protein that activates STAT3 [48]. IL6, as a multifunctional pro‐inflammatory cytokine, is an integral component of the intracellular feedback loop between tumor and host immunity, which regulates cancer progression through a variety of mechanisms, including accelerating EMT, stimulating angiogenesis, self‐renewal of CSCs, and facilitating tumorigenesis and increased metastasis [49, 50, 51, 52], and elevation of IL6 levels serves as a trigger of the JAK/STAT3 signaling pathway. MCPIP1 can physically interact with the stem‐loop structure in the 3′ UTR of transcripts through its structural domain PIN, leading to the destabilization of IL6 mRNA and then the degradation of IL6 [17]. Consistently, protein interactions between MCPIP1 and IL6 were also verified in the String database. Therefore, we investigated whether the effects of MCPIP1 on tumor EMT and tumor stemness were mediated by targeting the IL6/JAK2/STAT3 signaling pathway. A recent study showed that ISG15 can promote proliferation and metastasis through IL6/JAK2/STAT3 signaling in renal cell carcinoma [53]. Our findings suggest that MCPIP1 significantly inhibits stem and mixed EMT‐related growth, invasion, and extensive metastasis of PC cells through inhibition of the IL6/JAK2/STAT3 signaling pathway.

The main clinical challenges in pancreatic cancer are early diagnosis difficulty, therapeutic resistance, and poor prognosis. Our experiments show that MCPIP1 expression in PC tissues is significantly lower than in adjacent normal pancreatic tissues. Additionally, MCPIP1 inhibits cancer stemness and mixed EMT of PC cells by suppressing the IL6/JAK2/STAT3 axis. In PC xenograft models, lentivirus‐mediated MCPIP1 knockdown led to larger‐sized tumors versus controls. In Gem‐resistance experiments, MCPIP1 enhanced the sensitivity of PC cells to Gem. These findings indicate that low MCPIP1 expression promotes tumor progression and accelerates PC deterioration, whereas MCPIP1 itself inhibits tumor progression and reverses drug resistance—suggesting it may act as a tumor suppressor gene. However, the clinical significance of MCPIP1 in PC is still in the early stage of translation from basic research to clinical application. Several limitations deriving from this study should be considered: first, the small number of clinical cases collected requires further verification of the stability and universality of our findings; second, intervention methods remain immature, as MCPIP1 knockdown was performed in animal models and preclinical safety evaluation is lacking; third, its mechanism of MCPIP1 activity requires clarification, and whether MCPIP1 in PC is regulated by other molecules or exerts subtype‐specific differences may affect clinical application.

The core clinical significance of MCPIP1 in pancreatic cancer is that, as a “tumor suppressor gene”, its low expression is closely related to the appearance, metastasis, drug resistance, and poor prognosis of pancreatic cancer. Thus, the key to translating the MCPIP1 regulation into practical therapies lies in restoring or enhancing its tumor suppressor function. In this study, MCPIP1 acts as a positive regulator that inhibits the IL6/JAK2/STAT3 axis; thus it is important to focus directly on restoring/enhancing MCPIP1 function and indirect pathway intervention. Potential methods include local enhancement through gene therapy [54], small‐molecule agonists, and reducing MCPIP degradation, although gene therapy must address vector targeting and safety.

Based on the role of MCPIP1 as a positive regulator inhibiting the IL6/JAK2/STAT3 axis, we propose that its expression may help identify patient subgroups with high IL6/JAK2/STAT3 axis activity (i.e., pancreatic cancer patients with strong metastatic potential). Such tumors may activate this pathway due to the reduction in MCPIP1 levels, making them more likely to benefit from IL6/JAK2/STAT3‐targeted therapies.

Future plans include validating the diagnostic and prognostic value of MCPIP1 using larger clinical samples, and specifically analyzing the correlation between its expression and clinical outcomes in patients with PC to support its potential in patient stratification. Once its diagnostic/prognostic value is verified in large‐scale samples and safe, effective interventions are developed, MCPIP1 is expected to evolve from a “basic research molecule” to a “practical tool” for the clinical diagnosis and treatment of PC. Specific intervention methods include gene therapy to achieve local enhancement.

In summary, this study identified MCPIP1 as a promising therapeutic target for hybrid EMT and tumor stemness. High MCPIP1 expression was positively correlated with patient survival time and predicted a good prognosis for patients with PC. Mechanistically, this study demonstrated that MCPIP1 knockdown promotes cell migration, invasion, and maintenance of stemness through the IL6/JAK2/STAT3 axis in PC. Taken together, these findings indicate that MCPIP1 could serve as a promising biomarker for predicting distant PC metastasis and a target for personalized drug therapy, with the potential to prevent tumor metastasis and improve survival outcomes in patients with cancer.

## Author Contributions

**Xihui Ding:** investigation, validation, writing – original draft, resources. **Yingying Zheng:** writing – original draft, validation, investigation, conceptualization. **Min Liu:** methodology, formal analysis, visualization. **Fu Lai:** software, visualization. **Shiqi Liu:** data curation, project administration. **Qiuping Chen:** formal analysis. **Zihao Zhu:** methodology. **Huanzhong Liu:** supervision. **Xiaohui Li:** supervision. **Jinyong Xu:** supervision. **Rui Wang:** writing – review and editing. **Zhenhua Ren:** supervision, funding acquisition.

## Ethics Statement

The Research Ethics Commission of Anhui Medical University approved the study protocol (No. 20241477). All experimental procedures complied with the Institutional Ethics Committee‐approved protocol and the relevant ethical guidelines. When the tumor diameter in nude mice exceeded 1.5 cm and the tumor volume remained below 10% of the body weight, euthanasia was performed.

## Consent

This study was approved by the Research Ethics Commission of Anhui Medical University (No. 83244511). All participants provided written informed consent.

## Conflicts of Interest

The authors declare no conflicts of interest.

## Supporting information


**Figure S1:** cam471179‐sup‐0001‐FigureS1.docx.

## Data Availability

The data generated in the present study may be requested from the corresponding author.
