# Supplementary material for: MCPIP1 Controls Hybrid EMT and Tumor Stemness via the IL6/JAK2/STAT3 Axis in Pancreatic Cancer
Source: Cancer Med. 2025 Aug 28;14(17):e71179. doi: 10.1002/cam4.71179 (PMC12392288; doi:10.1002/cam4.71179)
Supplement: Supplementary file 1 — Figure S1: cam471179‐sup‐0001‐FigureS1.docx. [file CAM4-14-e71179-s001.docx]

FigS1.tif


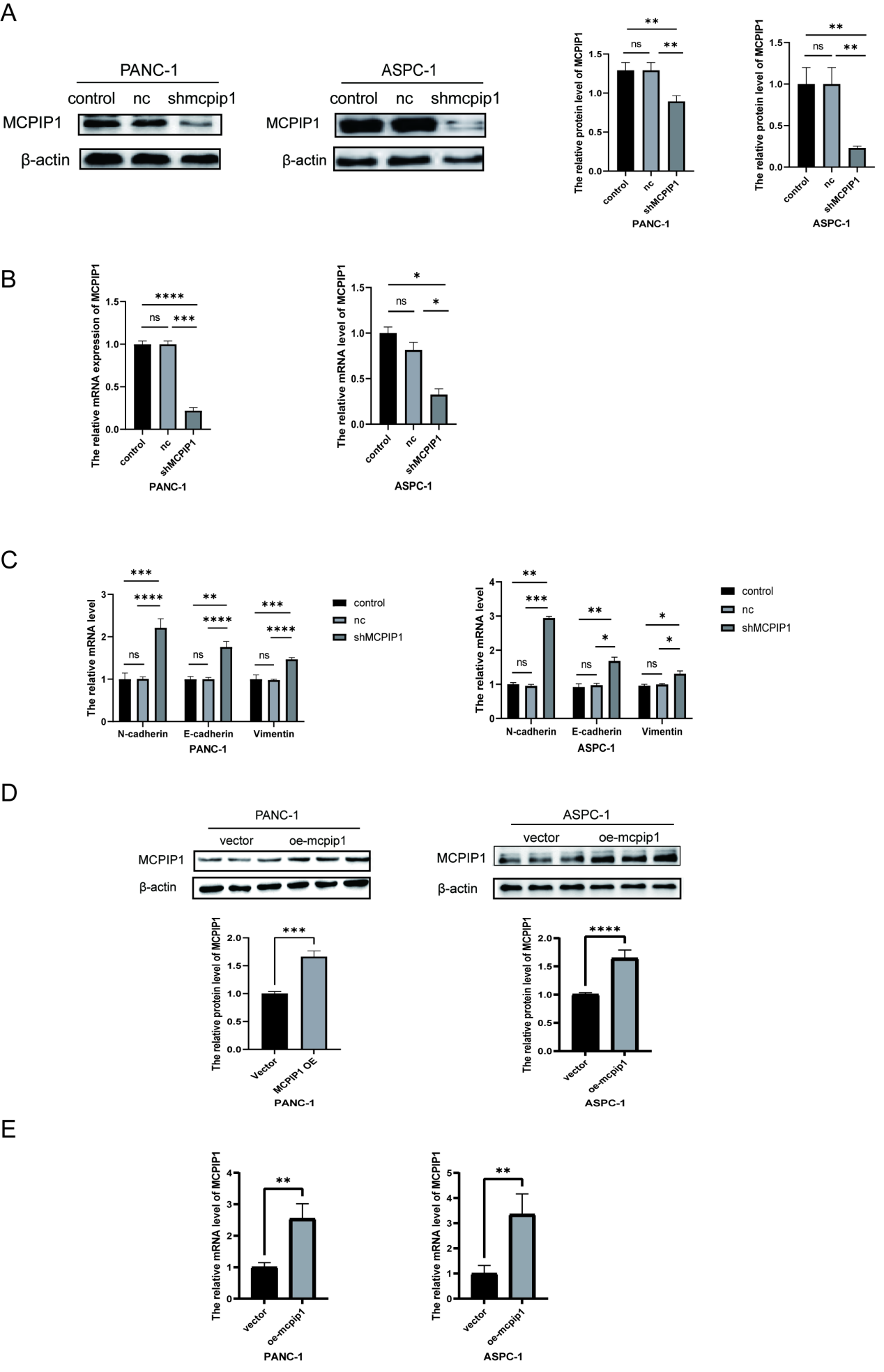


**Figure S1**. (A) MCPIP1 protein changes after down-regulation of MCPIP1 in tumor cells were analyzed by immunoblotting. (B) Enabling the analysis of MCPIP1 mRNA expression in pancreatic tumor cells by qRT-PCR. (C) Enabling the analysis of E-cad, N-cad, and Vimentin mRNA expression in pancreatic tumor cells by qRT-PCR. (D) MCPIP1 protein changes after up-regulation of MCPIP1 in tumor cells were analyzed by immunoblotting. (E) MCPIP1 mRNA expression in pancreatic tumor cells was assessed using quantitative reverse transcription PCR. Control, a group that has not received any treatment, nc, negative control group, shmcpip1, short hairpin RNA targeting mcpip1,vector, empty vector lentivirus, oe-mcpip1,overexpression of mcpip1. The mean ± S.D. of the three experimental groups is used to show the data’ significance, with * P < 0.05, ** P < 0.01, *** P < 0.001, and **** P < 0.0001 being their specific meanings.
